# The Potential of Liposomes with Carbonic Anhydrase IX to Deliver Anticancer Ingredients to Cancer Cells *in Vivo*

**DOI:** 10.3390/ijms16010230

**Published:** 2014-12-26

**Authors:** Huei Leng Helena Ng, Aiping Lu, Ge Lin, Ling Qin, Zhijun Yang

**Affiliations:** 1 School of Chinese Medicine, Hong Kong Baptist University, 7 Baptist University Road, Kowloon Tong, Kowloon, Hong Kong 999077, China; E-Mails: helena_ng@hkbu.edu.hk (H.L.H.N.); aipinglu@hkbu.edu.hk (A.L.); 2 Changshu Research Institute, Hong Kong Baptist University, Changshu Economic and Technological Development (CETD) Zone, Changshu 215500, China; 3 School of Biomedical Sciences, Chinese University of Hong Kong, Area 39, CUHK, Shatin, NT, Hong Kong 999077, China; E-Mail: linge@cuhk.edu.hk; 4 Musculoskeletal Research Laboratory, Department of Orthopaedics and Traumatology, The Chinese University of Hong Kong, Prince of Wales Hospital, Shatin, NT, Hong Kong 999077, China; E-Mail: qin@ort.cuhk.edu.hk

**Keywords:** immunoliposomes, anticancer ingredients, *in vivo* delivery, carbonic anhydronase IX (CA-IX), cancer cell penetrating peptides

## Abstract

Drug delivery nanocarriers, especially targeted drug delivery by liposomes are emerging as a class of therapeutics for cancer. Early research results suggest that liposomal therapeutics enhanced efficacy, while simultaneously reducing side effects, owing to properties such as more targeted localization in tumors and active cellular uptake. Here, we highlight the features of immunoliposomes that distinguish them from previous anticancer therapies, and describe how these features provide the potential for therapeutic effects that are not achievable with other modalities. While a large number of studies has been published, the emphasis here is placed on the carbonic anhydrase IX (CA-IX) and the conjugated liposomes that are likely to open a new chapter on drug delivery system by using immunoliposomes to deliver anticancer ingredients to cancer cells *in vivo*.

## 1. Introduction

Cancers are among the leading causes of death worldwide, accountable for 82 million (14.8%) of deaths in 2012 [1]. Despite extensive efforts made to improve the outcome of cancer therapy, cancer treatment is often limited by the therapeutic efficacy of anti-cancer agents. In many cases, anti-cancer agents are rapidly cleared from the circulation, or result in non-specific uptake by normal sensitive cells and tissues. One of the main focuses in current research is to develop targeted drug delivery systems that are able to enhance the treatment efficacy and reduce the side effects of anti-cancer agents in the clinical setting [2,3,4,5].

Nanocarriers created for therapeutics are comprised of therapeutic entities and components that assemble with therapeutic entities, such as lipids and polymers [6]. Lipidic drug carriers with water phase are generally called liposomes. While it may be argued that life forms may have never been developed without the protection of enclosing lipidic membranes, human cells are naturally and mainly comprised of lipid molecules. Hence the advancement in molecular lipid nanotechnology has permitted dramatic progress in human medical services.

Over the past 50 years, since the sealed phospholipids’ lamellae of liposomes were observed to be able to differentially impede the diffusion of ions, the study of organized assemblies of phospholipids has taken place in diverse fields including pharmaceutical science, pharmacology, and cell biology. Since the early 1980s, liposomes have gained substantial interest commercially; however, neither strategy for the advancement of technology nor any general accepted area for its practical uses were exploited for many years [7]. From the end of 20th century, liposomal and lipid-complexed products became commercially available and recognized for their clinical applications. Liposomal science has established itself as a commercially important discipline, and it has been propelled forward by the understanding of how individual molecules including active ingredients and pharmaceutical excipients (effectively targeting molecules) assemble into lipidic nanocarriers [7].

Firstly, in this review, we briefly summarize the development of liposomal therapeutics. Next we discuss the key challenges of liposomes for *in vivo* delivery of anticancer ingredients. Finally, we discuss the role of CA-IX as a molecular target for liposomal-based cancer therapy. The utmost difficulty for liposomes is accurate *in vivo* delivery of the anticancer ingredients into cancer cells in the tissues of the body. Thus, our emphasis here is on the liposomes targeted delivery of anticancer ingredients into cancer cells *in vivo*. Although there are many experimental approaches utilizing liposomes that can be affected by external stimulation, we focus on systemic administration of anticancer ingredients to the cancer cells by liposomes in the tissues of the body.

## 2. The Development of Liposomal Therapeutics

Liposomes were first discovered by Alec D. Bangham and R. W. Horne [8,9] in the early 1960’s. Liposomes are artificially self-assembled phospholipids mainly composed of a vesicle with a bilayer lamellar [10]. The integrity bilayer structure could release its contents after detergent treatment (structure-linked latency) [11]. Liposomes can be easily distinguished from micelles and hexagonal lipid phases by negative staining transmission electron microscopy [12]. In the presence of water, the phosphate heads of phospholipids are attracted to water, and line up to form a surface facing the water; the hydrocarbon tails of phospholipids are repelled by water, line up to form a surface facing the tails of another phospholipids; this combination forms a monolayer structure—micelle [13], or bilayer structures lamellar—liposomes [14]. Micelles and bilayers form in the polar medium by a process known as the hydrophobic effect [15]. One layer of hydrophilic heads faces outside of the liposomes, attracted to the water in the environment. Another layer of hydrophilic heads faces inside the liposomes, attracted by the water inside the liposomes [16]. Inside the hydrophobic membrane, liposomes encapsulate an aqueous phase of hydrophilic anticancer ingredients which cannot freely go through the lipids. On the other hand, hydrophobic chemicals can be inserted into the membrane. In this way, liposomes can carry both hydrophobic and hydrophilic molecules, and can be designed to deliver anticancer ingredients *in vitro* and *in vivo*. Intracellular delivery of anticancer ingredients encapsulated in the liposomes is mediated by endocytosis.

Research on liposomes has progressed from the first generation conventional vesicles to the second generation liposomes, in which long-circulating liposomes are obtained by modulating lipid composition, size, and charge of the vesicle. Liposomes may contain lower (or higher) pH aqueous anticancer ingredient solutions, and the anticancer ingredient will be neutralized to freely pass through the membrane. The pH changes can induce multilamellar vesicles to reassemble into unilamellar vesicles [17]. This process with electrostatic phenomenon was named as pH-induced vesiculation. On the other hand, pH and/or ion changes can also form a pH-gradient and or ion-gradient within the liposomes to increase the encapsulation of some electrolytic anticancer ingredients [18,19,20]. Generally, an acidic intra-liposomal aqueous environment was created using ammonium sulphate solution [21,22]. This approach allows protonation of the drug (e.g., gemcitabine) and prevents the back-diffusion of the drug from the liposome, resulting in an encapsulation efficiency of ~90%.

Additionally, polyethylene glycol (PEG) conjugated on the liposomal surface [23] allows liposomes to avoid detection by the reticuloendothelial system and other immune system, and show a longer blood-circulation time while reducing mononuclear phagocyte system (MPS) uptake. However, studies reported that PEG does not improve cellular uptake of liposome [24,25]. One hypothesis behind this was that the presence of PEG on the liposome surface not only prevents the MPS uptake, but also hinders the interaction of liposomes with cells and impedes the entry of the liposome into tumour cells [24]. In fact, how much PEG coating is required to increase systemic circulation without affecting the binding of liposome to tumor cell is not known. Interestingly, following the modification of the terminal PEG molecule, stealth liposomes can be linked with ligands that are expected to deliver pharmaceutical agents for cancer and other diseases. These ligands could be monoclonal antibodies [26,27,28,29], vitamins [30,31,32], or specific peptides [33,34].

## 3. Key Challenges of Liposomes for *in Vivo* Delivery of Anticancer Ingredients

Over the years, liposome-based anticancer ingredient delivery system has been extensively researched to improve pharmacotherapy. The major challenge for liposome-based cancer therapy is to increase anticancer ingredient delivery to tumour tissues while minimizing anticancer ingredient toxicity in normal tissues. Liposomes are known for their biodegradability, biocompatibility, and flexibility in structure [35,36,37]. Several anticancer ingredients with liposomal formulation have been developed, including cisplatin [38], cytarabine [39], daunorobucin [40], doxorubicin [41], methotrexate [42], paclitaxel [43], gemcitabine [4,5,44,45], and vincristine [46]. The liposomal doxorubicin (Doxil^®^, Janssen Biotech, Inc., Horsham, PA 19044, USA), is the first successful FDA (U.S. Food and Drug Administration)-approved liposomal therapy for cancer treatment [20,47,48,49], including ovarian/breast cancer, Kaposi’s sarcoma, and multiple myeloma. As compared to the free anticancer ingredient, the liposomal-formulated doxorubicin exhibits enhanced tumour targeting and reduced systemic toxicity [20,50,51]. Recently, a meta-analysis [52] reported that liposomal-based therapy, in particular liposomal formulation of anthracyclines, had demonstrated lower toxicity incidence with better cardiac safety in randomised controlled trials as compared to the conventional anthracyclines treatment. Anthracyclines are cardio toxic drugs that in higher cumulative doses are associated with increased cardiotoxicity risk [53]. Using the liposomal formulation (including those with pegylated liposomes), the overall cardiac heart failure rate has been reduced [52]. Nonetheless, the natural properties of conventional liposomes have both pros and cons for anticancer drug delivery, and these are described in this section.

The first important property of liposomes is the anticancer ingredient release rates from liposomes. Anticancer ingredients encapsulated in liposomes are sometimes not bioavailable because they are not released from the liposomes with appropriate rates [54,55]. In contrast, liposome-based formulations for oral delivery can prolong the therapeutic effect of water soluble active pharmaceutical ingredients (API) by sustained release and controlled absorption [56].

The second key feature is the clearance of liposomes. Upon the administration of liposomes *in vivo*, liposomes interact with serum opsonins through opsonisation, resulting in the recognition and clearance of liposomes by the MPS in the liver and spleen [57,58]. Although the clearance of liposomes and the encapsulated anticancer ingredients from the circulation is via the cells of MPS, the interaction of liposomes with plasma proteins is determined mainly by physicochemical properties of the liposomes [59]. Several different strategies have been developed to increase the circulation time of liposomes by coating the surface of the liposomes with inert molecules, such as monosialoganglioside [60,61] and PEG [62,63], to form a spatial barrier [48]. Monosialoganglioside or PEG occupies the space immediately adjacent to the liposomes surface, which tends to eliminate other macromolecules from this periliposomal space [49]. As a result, interaction of liposomes with serum opsonins is hindered, and thus interactions of MPS with such liposomes are impeded. Anticancer ingredients encapsulated in liposomes have substantial changes in the pharmacokinetics and biodistribution as they adopt the pharmacokinetics of the liposomes [64].

The third feature is the passive targeting of liposomes. In a healthy blood vessel, endothelial cells are bound together by tight junctions and form a monolayer of cells that lines the endothelium; this serves as a barrier to impede any large particles in the blood from leaking out of the vessel. In contrast, endothelium of tumour blood vessels is lined by defective endothelial cells. The tumour endothelial cells are not tightly bound together, resulting in large fenestration and loss of their normal barrier function [65]. Liposomes with sizes less than 400 nm can enter tumour tissues via the leaky tumour vasculature, but are kept within the bloodstream by the endothelial wall of healthy tissue vasculature [66]. This ability is named as the enhanced permeability and retention (EPR) effect. However, this passive targeting property of the current liposomal-based treatment has several limitations. Firstly, the permeability and porosity of tumour vessels varies with the type and stage of tumours [67]. Therefore, the effect of passive targeting may not be feasible in all tumours. Furthermore, homogenous targeting of tumour cells within a tumour is limited by the efficiency of anticancer ingredient diffusion [68]. Nonetheless, the current clinical use of liposomal-based therapies does not exhibit specific anticancer ingredient targeting at cellular level [51,68].

A fourth important feature of liposomes is the intracellular delivery of anticancer ingredients. While liposomes are large hydrophilic structures and not easy to fuse with cell membranes, anticancer ingredients are delivered into cells via endocytosis [69]. However, the endocytosed liposomes together with the encapsulated anticancer ingredients are often digested within endosomes by lysosomal enzymes before eliciting any biological effects [70]. Recently, ligands were conjugated to the liposomes surface to draw forth internalization of liposomes and their contents into cancer cells [71,72]. Many other strategies have also been proposed to enhance the endosomal escape of liposomes, such as cationic lipids [73] and pH-sensitive peptides [74] that can enhance the delivery of liposomes. Furthermore, incorporation of fusogenic lipids or cell penetrating peptides (also named membrane active peptides) may increase cytoplasmic delivery [75].

Last but not least, a fifth significant consideration for liposomes is the diversity of cancer cells. To deliver anticancer ingredients to cancer cells, liposomes face the difficulty of cancer cell diversity. Cancer may occur by a single cell, which unchecked, reproduces aggressively and invades many other tissues, and chaotically destroys normal tissue cells. The wider the cancer spreads, the harder it becomes to eradicate.

With the recent advances in molecular technology, anticancer ingredient-loaded liposomes could be actively targeted at the cellular level by designing ligand-targeted immunoliposomes. This can be achieved by incorporating the targeting ligands, such as proteins, peptides, monoclonal antibodies, fragment antigen-binding (Fab), and single-chain variable fragment (scFv) [50,76], onto the surface of liposomes to specifically target tumour cells that overexpress a particular cell surface antigen or receptor. The use of antibodies conjugated to liposomes in order to confer target specificity was first introduced in the 1980s [77,78]. The molecular- or receptor-targeted immunoliposomes have since gained extensive attention as the new generation of targeted anticancer ingredient delivery system.

## 4. The Rising of CA-IX as a Targeted Anticancer Drug Delivery System

In the treatment of diverse cancers, anticancer ingredients such as Doxorubicin (Doxil), Camptothecin and Daunorubicin (Daunoxome) are marketed in liposome delivery drugs. Although the interesting property of liposomes is their natural ability to target some cancer related tissues, their *in vivo* targeting effectiveness is still perplexing. At this stage, the ideal liposomes are those that can distinguish between normal cells and cancer cells. Nevertheless, no such functional liposomes have been developed thus far. However, the CA-IX [23], carbonic anhydrase XII (CA-XII) [79] and cancer cell penetrating peptides [80] may help liposomes to overcome the diversity of cancer cells in order to deliver the anticancer ingredients to kill most of the cancer cells but not normal cells. In this review, the following discussion about CA-IX may provide some encouragement to pharmaceutical scientists to develop liposome-mediated targeted drug delivery therapeutics for cancer patients.

Carbonic anhydrases (CAs) are a family of zinc metalloenzymes with 16 isoforms [81,82]. CAs are commonly known for their catalytic activity in the reversible hydration of cell-generated carbon dioxide into bicarbonate ions and protons (CO_2_ + H_2_O ↔ HCO_3_^−^ + H^+^). This catalytic reaction is an important event involved in many physiological and pathological processes, including respiration and transport of CO_2_ and HCO_3_^−^ between metabolizing tissues and lungs, electrolyte secretion in various tissues, homeostasis of pH and CO_2_, several biosynthetic reactions (e.g., gluconeogenesis, lipogenesis and ureagenesis), bone resorption and calcification, and tumorigenicity [82,83,84].

Of all the CAs, CA-ΙΧ and CA-XII are overexpressed in many tumour types [82] and are associated with cancer progression, metastasis, and therapeutic response [81,85]. Both CA-ΙΧ and CA-XII are transmembrane proteins with an extracellular active site [86,87]. However, CA-ΙΧ has higher extracellular catalytic activity than CA-XII [82,88]. Several unique features of CA-ΙΧ has made it a suitable candidate for antigen-targeted drug delivery system in cancer therapy, including its structure, protein expression, and function in tumour cells.

### 4.1. Biochemical Structure of CA-ΙΧ

**Figure 1:**
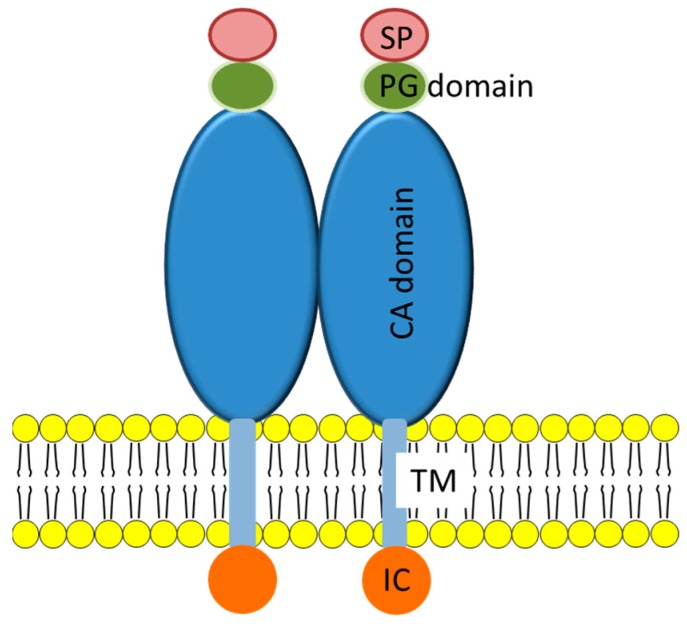
Dimeric structure of CA-ΙΧ. SP, signal peptide; PG, proteoglycan-like segment; CA, carbonic anhydrase domain; TM, transmembrane anchor; IC, intracytoplasmic tail [92].

CA-ΙΧ protein consists of a long extracellular CA domain, a transmembrane region, and an intracellular C terminus (Figure 1) [89]. Besides that, the proteoglycan-like domain which lies in between the signal peptide and the CA domain has been suggested to play an important role in promoting efficient CO_2_ hydration at the acidic micro-environment of hypoxic tumours [90,91]. This proteoglycan-like domain is a unique feature of CA-ΙΧ which is absent from other α-CAs. Localization of CA-ΙΧ on the tumour cell surface making it accessible to immunoliposomes, promotes its use as a candidate for targeted-drug delivery.

### 4.2. Functions of CA-ΙΧ in Tumour Tissue

Hypoxia is the key driver of CA-ΙΧ expression in tumour cells, and is induced by an HIF-1α-mediated signalling cascade [82,85]. Activation of HIF-1α (Hypoxia-inducible factor 1-α) leads to a glycolytic switch in the tumour cell metabolism pathway, resulting in increased production and export of lactic and carbonic acids to the extracellular space, and leads to a decline in extracellular pH (pHe). This acidified extracellular space can disrupt the intracellular pH (pHi), which will in turn affect basic cell function [81]. However, CA-ΙΧ is the key defensive player of the pHi. The overexpression of CA-ΙΧ during hypoxia catalyzes the hydrolysis of CO_2_ to HCO_3_^−^ and H^+^ in the extracellular microenvironment [81,93,94]. HCO_3_^−^ is actively transported via the sodium bicarbonate co-transporter into the cancer cell, thereby buffering pHi and maintaining cell survival [81,93,94]. On the other hand, the H^+^ remains extracellularly, generating an increasingly acidic microenvironment that promotes tumour cell invasiveness [81,93,94]. Importantly, CA-ΙΧ has a maximal catalytic activity at pH values of 6.49, typical of the acidic microenvironment of the hypoxic solid tumours in which CA-ΙΧ is overexpressed [91].

In addition to the regulation of intratumoral pH and tumour cell survival, studies [84,95,96,97] suggest that CA-ΙΧ regulates cell adhesion, migration and proliferation, indicating that CA-ΙΧ might enhance the metastatic potential of tumour cells. E-cadherin is a key regulator of cell-cell adhesion in epithelial tissues, in which the loss or destabilization of its function is linked to tumour invasion. Švastová *et al.* [97] showed that CA-ΙΧ competed with E-cadherin to modulate E-cadherin-mediated cell adhesion via interaction with β-catenin. CA-ΙΧ-expressing Madin-Darby canine kidney (MDCK) cells displayed a higher degree of cell dissociation as compared to normal MDCK cells, and the co-precipitation of CA-ΙΧ with β-catenin was indicated to be responsible for the lower cell adhesion capacity [97]. CA-ΙΧ-transfected C33A cell line (a human cervical carcinoma cell line) exhibited reduced cell-cell adhesion and increased cell motility via Rho-GTPase-mediated epithelial-mesenchymal transition [84]. Furthermore, CA-ΙΧ has also been reported to promote cell proliferation in renal cell carcinoma [98], cervical cancer cell [98], colorectal tumours [95], and gastric adenocarcinoma (AGS (the human gastric epithelial) cell line) [99].

### 4.3. Protein Expression of CA-ΙΧ

CA-ΙΧ is overexpressed in many solid tumours but not in their corresponding normal tissues [100]. Previous studies reported that CA-ΙΧ expression was up-regulated and associated with poor prognosis in cancers of the lung [101,102,103], breast [104,105,106], liver [107,108], cervix [109,110,111,112], colon [95,113], ovaries [114], bladder [115], head and neck [116,117,118], brain [119,120], and oral cavity [121,122]. Importantly, CA-ΙΧ expression in normal tissue is limited to the basolateral surface of gastric, intestinal (proliferating crypt enterocytes of the duodenum, jejunum and ileal mucosa), and gallbladder epithelia in human [96,123].

A study by Gut *et al.* [124] showed that mice with global knockout (KO) of CA-ΙΧ developed gastric hyperplasia of the glandular epithelium with numerous cysts. Nonetheless, the CA-ΙΧ KO mice developed normally and showed normal levels of gastric pH, acid secretion and serum gastrin [124]. Given that there are 16 isoforms of CAs, there could be a functional redundancy amongst the CA isoforms. It is also important to note that CA-ΙΧ deficiency in mice did not promote tumorigenecity [125].

Differential expression of CA-ΙΧ in normal *vs.* tumour cells can be explained by the mechanism of transcription for CA-ΙΧ. In the promoter region of the CA-ΙΧ gene, a hypoxia responsive element is located adjacent to its transcriptional start site [126]. The hypoxia responsive element in the CA-ΙΧ gene promoter binds to hypoxia-inducible factor 1α (HIF-1α), and this is expressed in many tumour types, overlapping with vascular endothelial growth factor (VEGF) mRNA and the hypoxia marker pimonidazole [126]. Interestingly, the promoter region of CA-ΙΧ gene has neither a TATA box nor a consensus initiator motif [89], suggesting that CA-ΙΧ is tightly regulated by hypoxia, and that HIF-1α may interact directly with the basal transcriptional machinery operating on this gene [126].

Hypoxia is a salient feature of rapidly growing malignant tumours and their metastases. During the early stage of tumour development, tissue hypoxia occurs due to insufficient blood supply [127]. However, angiogenesis and neovascularization during tumour growth do not improve the tissue hypoxia status as these fail to provide adequate oxygen supply to the tumour [127]. Reduced oxygen availability in tumour tissue leads to the activation of a core cellular response to hypoxia, the transcription factors HIF-1α [128]. Conversely, in normoxia tissues, HIF-1α is modified by oxygen-sensitive prolyl hydroxylases and asparaginyl hydroxylase, making it recognisable by the tumour suppressor von Hippel Lindau protein, resulting in the suppression of CA-ΙΧ expression [88].

It is well-accepted that one-size-fits-all approach is not applicable for cancer treatment. For example, triple-negative breast cancer, which accounts for about 23% of all breast cancer cases [129], is defined as lacking expression of oestrogen receptor, progesterone receptor, and HER2. Thus, HER2-targeted treatment is not suitable for triple-negative breast cancer cases. Importantly, about 43% of HER-2 negative breast cancer type expressed CA-IX [130], and CA-IX expression is associated with shorter relapse-free survival and worse survival [131]. Table 1 lists some examples of the differential tissue expression of HER2 and CA-IX in various tumour tissues from patients. This table indicates that, in some cancer types, HER2 might be a better molecular target than CA-IX for anticancer drug delivery, while in other cancers, CA-IX may be a better molecular target. Therefore, the most studied HER2-targeted treatment might only be beneficial for a small population of cancer patients.

**Table 1:** Examples of tissue expression of HER2 and CA-IX in various carcinomas.

Carcinoma	HER2 Expression	CA-IX Expression
Bladder micropapillary carcinomas typical urothelial carcinoma	15% of 61 [132] 9% of 100 [132]	N.D. 71% of 340 cases [133]
Brain	N.D.	97% of 112 cases [120]
Breast	18% of 1134 cases [129]	30% of 740 cases [130]
Cervix	14% of 50 cases [134]	82% of 221 cases [112]
Colorectal	4% of 51 cases [135]	49% of 80 cases [113]
Endometrial	12% of 286 cases [136]	89% of 92 cases [137]
Gastric	12% of 131 cases [138]	48% of 42 cases [139]
Gastroesophageal	24% of 100 cases [138]	49% of 39 cases [139]
Head and neck	7% f 57 cases [140]	26% of 72 cases [141]
Kidney clear cell renal cell carcinoma	Detected in normal tissue Rare [142]	99% of 186 cases [143]
Liver	N.D.	30% of 69 cases [108]
Lung	13% of 563 cases [144]	82% of 175 cases [103]
Oral cavity	1% of 196 cases [145]	43% of 80 cases [122]
Ovarian	29% of 50 cases [146]	18% of 205 cases [114]
Prostate	14% of 216 cases [147]	0% of 59 cases [148]

Expressions were detected with immunohistochemistry, enzyme-linked immunosorbent assay, Western blot, or fluorescence *in situ* hybridization assays. N.D., not determined.

### 4.4. CA-ΙΧ-Targeted Therapeutic Approaches

Binding of antibodies to cell surface antigens is able to lyse cells by complement activation or by antibody-mediated cell cytotoxicity (ADCC). Direct binding of the monoclonal antibody (mAb) to CA-ΙΧ can elicit an anti-tumour response due to ADCC. G250 is an IgG mAb that was first discovered in the 1980s, it was originally isolated from a hybridoma produced from splenocytes of mice following immunization with human renal-cell carcinoma cells [149]. G250 mAb has since been shown to recognize a conformational determinant of CA-ΙΧ [150,151]. However, G250 is not capable of inducing ADCC in renal-cell carcinoma cells. Instead, a human/mouse chimeric version of G250, cG250 (mouse variable fragment (Fv) and human IgG1 fragment crystallizable (Fc)), has been developed [152]. Studies [152,153] showed that cG250 could initiate cell lysis through ADCC in CA-ΙΧ-positive cells and that cG250-induced ADCC was significantly enhanced by co-treatment with interleukin 2 (IL-2). cG250 (Girentuximab) is currently marketed by WILEX AG (Munich, Germany) under the trade name RENCAREX^®^. Phase I, II and III clinical trials demonstrated that Girentuximab was safe, well tolerated, and demonstrated clinical benefit and disease stabilization alone [154,155,156] and together with IL-2 [157,158] or interferon (IFN)-α [159] treatment. Reports showed that most, if not all, adverse effects developed during the treatment period were attributable to the administration of IL-2 and IFN-α [158,159]. However, phase III clinical trial of Girentuximab in the treatment of clear cell renal cell carcinoma showed no improvement disease free survival rate [154].

Zaťovičova *et al.* [160] recently characterised a new CA-ΙΧ-specific mouse mAb VII/20. VII/20 mAb was directed to the catalytic domain of CA-ΙΧ, in which the receptor-mediated internalization was induced upon the binding of VII/20 mAb to CA-ΙΧ. Although *in vivo* study showed that VII/20 limited tumour growth in HT-29 colorectal xenografts, the effect was modest when tumours were established prior to treatment [160]. However, incorporation of VII/20 mAb onto liposomes might facilitate drug delivery to CA-ΙΧ-positive tumour cells and thereby enhance therapeutic output. On the other hand, Bayer HealthCare AG, Berlin, Germany [161] identified a 3ee9 mAb that selectively co-immunoprecipitated with and internalized by CA-ΙΧ-positive cells. The therapeutic application of this antibody has thus far been tested with monomethyl auristatin E as an antibody-drug conjugate (BAY79-4620) in several preclinical human xenograft tumour models [161]. Nonetheless, the development and characterization of new CA-ΙΧ-specific antibodies is still ongoing.

### 4.5. Optimization of Antibody-Targeted Immunoliposomes

PEG has been widely used as polymeric steric stabilizer. It is particularly useful because of its ease of preparation, relatively low cost, its molecular weight and structure can be modulated, and it serves as a linker to lipids or protein [48,162]. The most commonly used method to incorporate PEG in the liposomal membrane is via a cross-linked lipid (*i.e.*, distearoylphosphatidylethanolamine (DSPE)-PEG. DSPE-PEG containing a sulfhydryl-reactive group, such as maleimide, readily reacts with the thiol group of cysteine or the reduced thiol group of Fab or scFv fragments.

**Figure 2:**
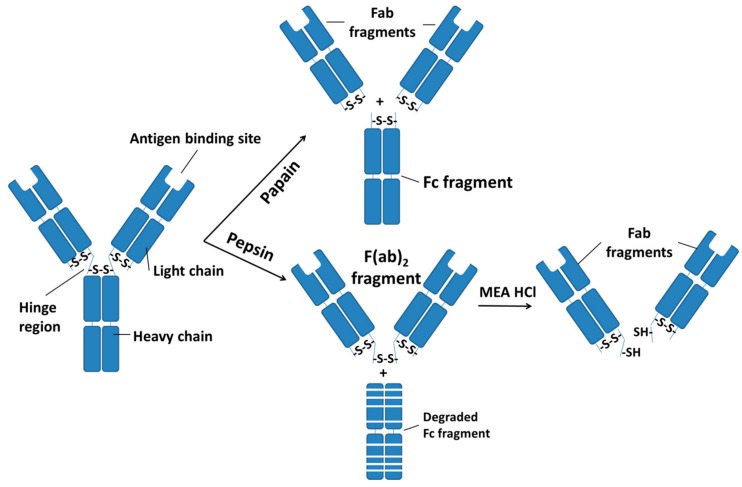
Introduction of sulfhydryl group in IgG antibodies (such as G250 mAb) via enzymatic digestion. Enzyme digestion of IgG antibodies with papain results in cleavage of the hinge region above the interchain disulfides. This produces two identical Fab fragments (heavy-light chain pairs), each containing one antigen binding site and one Fc region. Pepsin digestion of IgG antibodies cleaves below the disulphides in the hinge region, resulted in the formation of a bivalent fragment F(ab)_2_. The remaining Fc region is extensively degraded into smaller peptide fragments. F(ab)_2_ fragments are subsequently reduced with MEA-HCl and produced two Fab fragments.

Using G250 as an example, G250 can be modified for the conjugation onto the liposomes. G250 is an IgG molecule which is composed of two light and two heavy chains, held together by non-covalent bonding as well as a number of disulfide bonds. The hypervariable sequence in the *N*-terminal of the heavy—light chain proximity on each of the basic IgG-type monomeric structures are antigen binding sites (Figure 2) [162,163]. One of the methods to modify G250 mAb is enzyme digestion, as illustrated in Figure 2. Enzymatic digestion with papain produces two Fab fragments of the IgG molecule, each containing an antigen binding site, and one larger Fc (fragment crystallizable) fragment containing only the lower portions of the two heavy chains [164]. Otherwise, pepsin cleavage produces one large F(ab)_2_ fragment containing two antigen binding sites and many smaller peptide fragments from extensive degradation of the Fc region [164]. Specific reduction of the disulphide bonds which hold the F(ab)_2_ fragment together using 2-mercaptoethylamine-HCl (MEA-HCl) produces two Fab fragments, each of which has one antigen binding site. The advantage of using a Fab fragment instead of whole body antibody is the removal of the Fc region during enzymatic digestion, in which the immunogenic effect of the Fc portion increased reticuloendothelial system clearance through specific recognition by phagocytic cells carrying Fc receptor [162]. Additionally, the thiol group of the Fab fragment is readily bound with the maleimide of the DSPE-PEG to form an immunoliposomes.

## 5. Immunoliposome

One of the most studied ligand-targeted immunoliposomes was the human epidermal growth factor receptor 2 (HER2)-targeted immunoliposomes. HER2 is overexpressed in SKBR-3 or BT-474 breast cancer cells *in vitro* [27], and it is overexpressed at 1000-fold higher than normal in ~25% of breast cancer cases [165,166]. Most [27,28,167,168], but not all [169] studies reported significant enhanced anti-tumour efficacy with the HER2-targeted immunoliposomes. Park *et al.* [28] showed that HER2-targeted doxorubicin liposomes with internalizing ligand exhibited a significant increase in anti-tumour efficacy and reduced systemic toxicity compared with the non-targeted doxorubicin liposomes. However, the therapeutic efficacy of the non-targeted doxorubicin liposomes was not different from that of the HER2-targeted doxorubicin liposomes without the internalizing ligand [169]. This highlights the importance of the target antigen selection, as well as the importance of liposome internalization during the design of the immunoliposomes. Other antibodies used to target liposomes include: anti-CD22 antibody targeting Non-Hodgkin’s Lymphoma [170]; anti-CD19 antibody targeting malignant B cells [171]; anti-CD44 antibody targeting cancer stem cells [45,172]; and anti-MT1-MMP (membrane type 1 matrix metalloproteinase) targeting tumour and neovascularity [173].

Recently, a doxorubicin-loaded anti-EGFR (epidermal growth factor receptor) immunoliposome (anti-EGFR ILs-dox) phase I clinical trial for patients with EGFR-overexpressing advanced solid tumours was completed [174]. In this phase I clinical trial, Fab fragment of cetuximab, an EGFR mAb, was conjugated onto PEGylated liposomes containing doxorubicin. The primary end point of this study was the maximum tolerated dose of anti-EGFR ILs-dox, and it was reported that anti-EGFR ILs-dox was well tolerated up to 50 mg doxorubicin per m^2^. The best response to the six-month treatment included one complete response, one partial response, and ten stable disease lasting 2–12 months (median 5.75 months). A phase I clinical trial for MCC-465, a GAH-targeted doxorubicin-loaded immunoliposome, has also been completed [175]. In this study, patients with metastatic or recurrent stomach cancer were recruited. Maximum tolerated dose was 32.5 mg doxorubicin per m^2^ for a 3-week schedule. Although no antitumor response was observed, stable disease was observed in 10 out of 18 patients. Interestingly, no palmar-plantar erythrodysaesthesia, cardiotoxicity, or cumulative toxicity was observed in the patients in these studies, indicating immunoliposome had reduced systemic toxic side effects.

The development of ligand-targeted immunoliposomes is still at its preliminary stage, and these newly emerging drug delivery systems lack long-term results. With the extensive research in this area in the past, ligand-targeted immunoliposomes have shown convincing pre-clinical results with enhanced therapeutic efficacy over the passively-targeted liposomes [76,176]. Nonetheless, new challenges have arisen from these past ligand-targeted immunoliposomes design, such as internalization into the tumour cells, immune recognition, restricted diffusion and penetration through the tumour tissue, non-specific binding by serum proteins, toxic side effects of liposomal lipid composition, and translation from pre-clinical animal models to clinical studies [76,177]. In recent years, carbonic anhydrase IX (CA-ΙΧ) has drawn a lot of attention from researchers as a potential candidate for antigen-targeted anticancer ingredient delivery.

### 5.1. CA-ΙΧ-Targeted Immunoliposomes

The first CA-ΙΧ-targeted immunoliposomes was developed by Shinkai and colleagues [178] in 2001, In this study [178], magnetoliposomes (MLs) were conjugated with the Fab fragment of the G250 antibody to induce hyperthermia in cancer cells. MLs consist of magnetisable iron oxide cores (magnetite, Fe_3_O_4_) which can mediate a magnetic field-induced hyperthermia therapy. *In vitro* uptake of G250 MLs was five times higher in the CA-ΙΧ-expressed mouse renal-cell carcinoma cells, and was 1.5 times higher than the non-targeted MLs [178]. Furthermore, 50% of the injected G250 MLs accumulated in the tumour tissues; this was approximately 27 times higher than that of the non-targeted MLs [178]. Non-specific uptake of G250 MLs was mainly discovered in the blood and the liver, and this could be due to the uptake by macrophages and Kupffer cells [178]. However, the non-specific uptake of G250 MLs was lower than that of the non-targeted MLs, and accumulation of G250 MLs in the liver did not induce tissue necrosis. Treatment of G250 MLs in mice has significantly reduced the tumour tissue weight and increased the survival time of mice. This study presented a novel approach using CA-ΙΧ as the antigen targeted for liposome delivery and showed that CA-ΙΧ is a promising target for cancer therapy.

Recently, we developed a CA-IX-targeted immunoliposome delivery system for human lung cancer *in vitro* [26]. In this study, docetaxel was encapsulated in the CA-IX targeted immunoliposome and was delivered to human lung cancer cells *in vitro*. As compared to the non-targeted immunoliposome, CA-IX-targeted immunoliposome exhibited a 1.65-fold increase in binding affinity to CA-IX-positive human lung cancer cells. Docetaxel delivered via CA-IX-targeted immunoliposome induced a strong cytotoxicity effect in CA-IX-positive human lung cancer cells [26]. However, the effect of anti-cancer agents delivered via CA-IX-targeted immunoliposome has yet to be examined *in vivo*.

### 5.2. Limitations of CA-IX-Targeted Immunoliposomes

As mentioned above, the ideal liposomes are those that have target specificity for tumour cells but not to normal cells. However, such liposomes have not yet been developed. To date, the identified ligands for anticancer treatment do not embrace all cancer types. Moreover, ligands that have high ratio of tumour-to-normal tissue levels are essential to reduce side effects of cytotoxic drugs. CA-IX is expressed in many tumour types, thus it might serve as an additional cancer-specific ligand-targeted anticancer treatment. While CA-IX might not be the magic bullet for anticancer treatment of all cancer types, research on CA-IX targeted drug delivery system is warranted for a more comprehensive anticancer drug delivery system that could be beneficial for larger populations of cancer patients. Current research on CA-IX-targeted liposomal drug delivery system is however, very limited. CA-IX-targeted immunoliposome, theoretically, would be an ideal liposomal-based therapy for various types of cancer. However, more studies, including both *in vitro* and *in vivo*, are essential to prove the concept before moving on to clinical trials, Furthermore, CA-IX is expressed in the basolateral surface of gastrointestinal tissue, and gallbladder epithelia in human [96,123]. Therefore, it is crucial to determine the maximum tolerated dose of anticancer ingredients in cancer patients with minimum side effects. 

Furthermore, Askoxylakis *et al.* [179] suggested that expression of the CA-IX protein is unstable, and microenvironment-dependent. The scientific literature lacks information about the onset of CA-IX expression in the course of carcinogenesis, the percentage of CA-IX-positive cells, and the amount of protein expressed in tumour tissues at different disease stages. Thus, more research is needed in order to design a proper treatment regime for patients. The first identified peptide ligand for the CA-IX receptor through phage display technology is CaIX-P1 [179,180]. However, to the best of our knowledge, the endogenous ligand of the CA-IX receptor is unknown. Whether there will be an endogenous ligand present to compete with the CA-IX targeted immunoliposome for the CA-IX receptor, and thus limit the therapeutic outcome of CA-IX targeted immunoliposome, is currently unknown.

## 6. Conclusions

This review has focused on targeted liposomal technology and summarized CA-IX in relation to the principal liposomes formulations. CA-IX is a tumour-specific antigen for many tumour tissues, but not for their corresponding normal tissues. While current research on the CA-IX-targeted therapeutic approach has been mainly focused on human renal-cell carcinoma cells, this can certainly be expanded into other tumour types. To date, the therapeutic effect of CA-IX-targeted drug delivery to other tumour types has not been documented. Furthermore, research on CA-IX-targeted liposomal drug delivery system is very limited. Preclinical and clinical studies have demonstrated clear evidence that immunoliposomes exhibit specificity in the immune system with more powerful cytotoxics to enhance anticancer therapeutic efficacy and reduce side effects by targeted delivery, and thus warrant further research for clinical applications.
